# Development and Validation of a Self-reported Questionnaire for Measuring Internet Search Dependence

**DOI:** 10.3389/fpubh.2016.00274

**Published:** 2016-12-20

**Authors:** Yifan Wang, Lingdan Wu, Hongli Zhou, Jiaojing Xu, Guangheng Dong

**Affiliations:** ^1^Department of Psychology, Zhejiang Normal University, Jinhua, China; ^2^Department of Psychology, University of Konstanz, Konstanz, Germany; ^3^Institute of Psychological and Brain Sciences, Zhejiang Normal University, Jinhua, China

**Keywords:** Internet search dependence, questionnaire, psychological characteristic, validity, reliability

## Abstract

Internet search has become the most common way that people deal with issues and problems in everyday life. The wide use of Internet search has largely changed the way people search for and store information. There is a growing interest in the impact of Internet search on users’ affect, cognition, and behavior. Thus, it is essential to develop a tool to measure the changes in psychological characteristics as a result of long-term use of Internet search. The aim of this study is to develop a Questionnaire on Internet Search Dependence (QISD) and test its reliability and validity. We first proposed a preliminary structure and items of the QISD based on literature review, supplemental investigations, and interviews. And then, we assessed the psychometric properties and explored the factor structure of the initial version *via* exploratory factor analysis (EFA). The EFA results indicated that four dimensions of the QISD were very reliable, i.e., habitual use of Internet search, withdrawal reaction, Internet search trust, and external storage under Internet search. Finally, we tested the factor solution obtained from EFA through confirmatory factor analysis (CFA). The results of CFA confirmed that the four dimensions model fits the data well. In all, this study suggests that the 12-item QISD is of high reliability and validity and can serve as a preliminary tool to measure the features of Internet search dependence.

## Introduction

The advent of the Internet, with powerful and diverse search engines, has made it much easier now than in the past to access information. The high-speed Internet enables individuals to instantly exchange knowledge and information all over the world. As the Internet has become an essential tool in our daily life, it is important to notice that people are susceptible to the unprecedented Internet search environment (1–3). The majority of population spends many hours per day on the Internet and become increasingly dependent on the Internet search to meet various needs (3, 4). To date, there has been a growing interest in the affective, cognitive, and behavioral changes as a result of Internet search (5, 6), although empirical studies are still lacking. Further studies are needed to investigate the impact of Internet search on users’ psychological characteristics.

Previous studies have shown that the wide use of Internet search engines has largely modified the way human beings search for and store information. Sparrow et al. (7) found that the Internet search has become human beings’ primary form of external and transactive memory. Individuals are prone to turn to the Internet and search for answers online when facing difficult questions and controversial issues (8). People have gradually shifted from remembering the answers to searching for answers on the Internet and remembering where they could effectively find the right things (9). Consistently, a recent study found that Internet access reduced people’s feeling of knowing and lowered their willingness to voluntarily generate answers (10). Once the Internet is out of reach and when they cannot immediately find out what they want, people would show withdrawal-like symptoms, such as irritability, tension, and depression (11). Those symptoms are largely overlapping with withdrawal symptoms of substance addictions and pathological Internet use (12–14).

Nowadays more and more people are likely to make decisions or solve problems according to the suggestions and advices from Internet. A symbiotic relationship has been built up over the past years between people and the Internet (15). It becomes very common that people trust the online information blindly and are incapable of discriminating between reliable information and unreliable information (16). People have formed specific searching habits owing to long-term use of search engines. The more dependent on Internet search, the more keyword spotting, browsing, and scanning behaviors, and less contemplation would be detected among Internet users (17, 18). These findings indicate that Internet users may have developed certain search strategies to make the best use of the Internet rather than independent and in-depth thinking.

In all, previous studies have shown that Internet users are associated with various features of Internet search dependence. Future studies are needed to discern those features in details. Thus, developing a valid tool, such as a questionnaire, to measure people’s psychological characteristics of Internet search dependence is of great importance. This study aimed to develop a questionnaire with proper dimensions and good validity for measuring features of Internet search dependence, which we hope will be a practical tool for future studies to further explore Internet search dependence.

## Materials and Methods

### Step 1: Developing the Initial Questionnaire

The initial questionnaire consists of questions of basic information (such as gender, age, and history of Internet search) and 12 items about psychological characteristics related to the Internet search dependence, based on a comprehensive literature review, supplemental investigations, and interviews.

First, we queried several databases such as PubMed, ScienceDirect, Web of Science, and Springer Link with two keywords, Internet search and Internet use. Approximately 50 papers (1, 2, 5, 18, 19), published between year 2008 and year 2016, were carefully chosen to review.

Subsequently, we compiled 16 items to represent psychological characteristics associated with Internet search dependence, based on the literature review and a follow-up interview with 50 randomly selected university students (see Supplementary Material). We adopted the six criteria for behavioral addiction formulated by Griffiths (i.e., salience, mood modification, tolerance, withdrawal, conflict, and relapse) (20) and extracted the contents that are closely linked to Internet search. All the 16 items (see Supplementary Material) were then refined for the sake of unambiguity, simplicity, and clarity, which resulted in a 12-item questionnaire of Internet Search Dependence (QISD). This questionnaire has six dimensions covering all psychological characteristics relevant to Internet search dependence, i.e., trust in Internet search, withdrawal symptoms, search impulsivity, keywords spotting, decreased contemplation, and external storage. For each item, we used a five-point Likert-type scale, ranging from 0 (“Never”) to 4 (“Always”).

In addition, we conducted a supplemental investigation with 100 randomly selected university students (see Supplementary Material), through which we identified 5 extra characteristics of Internet search dependence. Those items were assigned to the section of “basic information” considering that they were mostly about the time and frequency of the Internet search (e.g., number of years that a person uses Internet search).

### Step 2: Exploratory Factor Analysis (EFA)

#### Participants

This research was approved by the Human Investigations Committee of Zhejiang Normal University. The initial version of the QISD was tested among a randomly selected sample of 320 university students from Zhejiang Normal University. The returning rate of questionnaires is 90.30%. Two hundred eighty-nine subjects completed the questionnaire (75 males and 214 females, the age ranged from 18 to 27 years, M = 22 years, SD = 1.98), while 31 subjects failed to finish the questionnaire and thus were excluded from data analysis. All subjects were promised anonymity and thanked with a pen or a mini notebook for their participation. All participants in this research signed a written informed consent and no vulnerable populations were involved.

#### Data Analysis

Psychometric properties, reliability and feasibility, were assessed by means of consistency coefficients (Cronbach’s α) and item analyses, respectively. Cronbach’s α is a measure of reliability, or internal consistency, of a set of items (21). Item analyses, which were composed of mean, standard deviation, kurtosis, skewness, critical ratio (CR), and item correlations, measure quality of individual items.

Exploratory factor analysis was implemented to investigate the construct validity of QISD by means of principle component analysis method. The analyses of Kaiser–Meyer–Olkin (KMO) and Bartlett’s test of sphericity suggested whether the data were suitable for orthogonal factor dimension (22). In addition, the principle component analysis was performed with Varimax rotation to facilitate interpretation of the factors. All analyses were performed using SPSS, version 22.

### Step 3: Confirmatory Factor Analysis (CFA)

#### Participants

The CFA was conducted among a newly randomly recruited sample of 324 university students to examine the dimensional structure and the validation of the revised QISD. Thirty-three incomplete questionnaires were excluded. The remaining 291 questionnaires obtained from 138 males and 153 females were used for data analysis. The mean age of the subjects was 22 years (SD = 2.14). All subjects were promised anonymity and thanked with a pen or a mini notebook for their participation.

#### Data Analysis

The CFA was carried out using maximum-likelihood estimation analysis in AMOS 21.0 (www.ibm.com/legal/copytrade.shtml) to verify the goodness of fit of the model that was built according to the results of EFA. Besides the χ^2^ and the χ^2^/df (cutoff ≤ 3) (23), other fit indices and incremental indices were also calculated, including the root mean square residual (RMR; cutoff ≤ 0.05), the goodness-of-fit index (GFI; cutoff ≥ 0.90) (24), the Tucker Lewis index (TLI; cutoff ≥ 0.90) (25), the comparative fit index (cutoff ≥ 0.90) (26), and the root mean square error of approximation (RMSEA; 0.05 < cutoff < 0.08) (27).

## Results

### Results of Item Analyses and Cronbach’s α

Table 1 shows the distribution of the item characteristics. The average of the total scores of the QISD was 25.81 (SD = 5.93). All items were statistically significant on CR (*p* < 0.01), suggesting that the discrimination of each item was good. Eight of the 12 items were right skewed (*g*_1_ > 0), ranging from 0.09 to 0.29; the remaining items were left skewed (*g*_1_ < 0), ranging from −0.46 to −0.26. The highest level of kurtosis and skewness were evident on the fifth item and the sixth item, respectively.

**Table 1:** **Item characteristics compose of critical ratio (CR), mean, skewness, kurtosis, and correlation coefficients of each item**.

Item	CR (*p*)	Mean (SD)	Skewness	Kurtosis	Correlation coefficient
1	−8.62**	2.94 (0.72)	−0.26	−0.19	0.54**
2	−10.20**	2.03 (1.09)	0.29	−0.60	0.52**
3	−6.64**	2.18 (1.01)	0.09	−0.68	0.42**
4	−8.31**	2.17 (0.97)	0.26	−0.42	0.51**
5	−8.36**	1.89 (1.15)	0.14	−0.80	0.53**
6	−10.11**	2.82 (0.82)	−0.46	0.03	0.59**
7	−8.18**	2.61 (0.82)	−0.20	−0.25	0.50**
8	−10.11**	1.75 (0.93)	0.11	−0.28	0.51**
9	−10.58**	1.58 (1.03)	0.11	−0.67	0.59**
10	−8.73**	1.80 (1.05)	0.26	−0.67	0.47**
11	−9.79**	1.69 (0.90)	0.21	−0.02	0.57**
12	−9.54**	2.35 (0.90)	−0.14	0.13	0.52**

**p<0.01.

Furthermore, the score of each item was positively related to the total score of QISD, with all the correlation coefficients greater than 0.40 (*p* < 0.01). The Cronbach’s α of QISD was 0.75, and the average inter-item correlation was 0.2 (ranging from 0.04 to 0.42), suggesting that the internal consistency is acceptable.

### EFA Results

**Figure 1:**
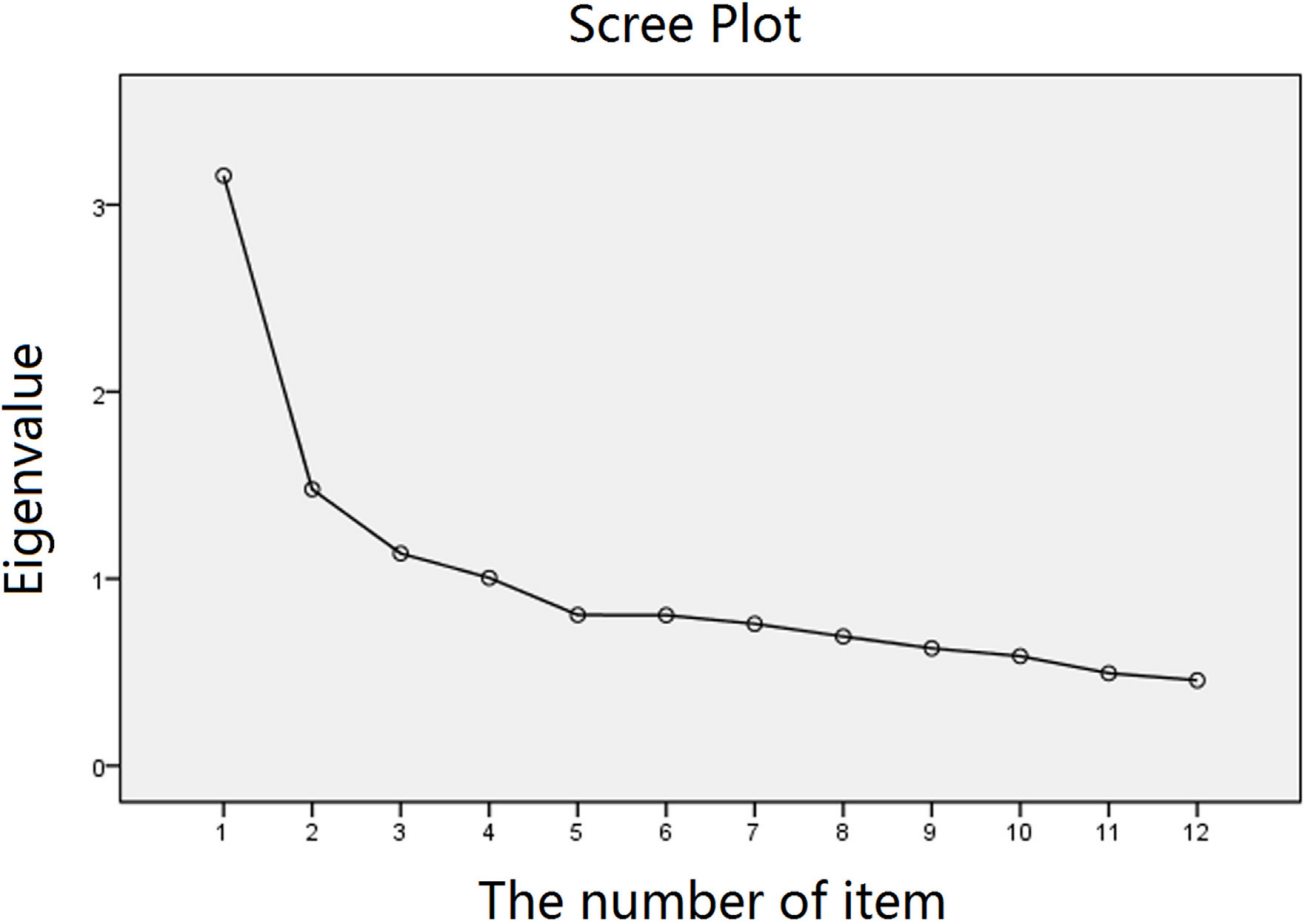
**The scree plot obtained from exploratory factor analysis**.

An EFA using principle component analysis was performed using SPSS to figure out the model that best fits the data. The value of KMO was 0.79, and Bartlett’s test met a significant level (χ^2^ = 561.19, *p* < 0.001), suggesting that the data were suitable for factor analysis. Following the recommendation of some relevant literatures, the dimensions of the model were extracted based on the percentage of variance, the scree plot, and the Kaiser–Guttman method (28). Accordingly, the factorial solution suggested a model with four dimensions that accounted for 55.92% of the total variance. The scree plot showed that the eigenvalues of the first four dimensions were greater than 1, and the eigenvalues tended to be equal after the fifth factor (Figure 1). In general, these results indicated that a large proportion e of the total variance was explained by the four factors.

The eigenvalues and corresponding percentage of variance accounting for each component are presented in Table 2, and factor loadings are presented in Table 3. The first dimension, “Habitual use of Internet search” (e.g., item 3, “try to abstract key words from a complex question”) measures the habits of Internet search. It is composed of item 1, 3, 4, 6, and 7. This dimension had an eigenvalue of 3.34 and accounted for 27.84% of the total variance. The second dimension, “withdrawal symptoms,” measures negative reactions when Internet search was not available (e.g., item 8, “I will be upset if I cannot use Internet search when encountering complex questions”). This dimension is composed of item 8 and 11 and had an eigenvalue of 1.28 and accounted for 10.70% of the total variance. The third dimension, “Internet search trust,” measures the degree of confidence to Internet search. It is composed of item 9, 10, and 12. This dimension had an eigenvalue of 1.08 and accounted for 8.99% of the total variance. The fourth dimension, “external storage under Internet search,” had an eigenvalue of 1.01 and accounted for 8.40% of the total variance. Each dimension was significantly correlated with the total score, and the correlation coefficients were greater than 0.6 (The Cronbach’s α coefficients for each dimension were 0.69, 0.67, 0.71, and 0.65).

**Table 2:** **Eigenvalues and corresponding percentage of variance explained**.

Eigenvalue	% of variance	Cumulative variance
3.34	27.84	27.84
1.28	10.70	38.53
1.08	8.99	47.53
1.00	8.39	55.92
0.91	7.60	63.52
0.8	6.71	70.23
0.76	6.32	76.55
0.7	5.79	82.34
0.63	5.28	87.61
0.53	4.40	92.01
0.51	4.24	96.26
0.45	3.74	100.00

**Table 3:** **The results of exploratory factor analysis: factor loadings**.

Item	Factor 1	Factor 2	Factor 3	Factor 4
3	0.71			
7	0.63			
6	0.56			
4	0.56	0.35		
1	0.50			
8		0.73		
11		0.69		
12			0.78	
10		0.42	0.56	
9			0.55	
2				0.83
5				0.79

According to the results of EFA, we revised the QISD by amending some sentences and words of the items for better comprehensibility. For example, the item 8, “I will be upset if I cannot use Internet search when encountering complex questions,” was modified as “I will be upset if I cannot find an answer to a complex question through Internet search.” In addition, we did not eliminate any items at this stage.

### CFA Results

Based on the aforementioned results, a four-factor model was further examined using CFA. The results of CFA are as follows: χ^2^ = 97.49, χ^2^/df = 2.03, *p* < 0.01; RMR = 0.045; GFI = 0.95; IFI = 0.91; TLI = 0.86; GFI = 0.90; RMSEA = 0.06. Although the TLI did not reach the cutoff value, it was quite close to 0.90. The results suggest that the four-factor model is a good fit to the data.

## Discussion

This study aimed to develop a psychometric tool to measure psychological characteristics of Internet search dependence and tested the validity and reliability of the questionnaire. The Cronbach’s α coefficient of reliability index was 0.75, suggesting that the questionnaire was reliable. Factor analyses revealed that the questionnaire contained four dimensions: habitual use of Internet search, withdrawal reaction, Internet search trust, and external storage under Internet search. Further analysis *via* CFA supported the four-factor model as most of the validity indexes were larger than the cutoff value. The results suggest that the QISD is valid and reliable.

Habitual use of Internet search implies the behavioral habits that people have cultivated during Internet search. Once people get used to Internet search, they gradually develop certain behavioral habits associated with Internet search when facing new and/or complex situations. For example, people are prone to abstract keywords from complex problems and seek for answers *via* Internet search when facing disputes or new questions (17).

The withdrawal symptoms refer to the response to a sudden abstinence from Internet search. Since Internet search has become a part of people’s daily routine, people may exhibit negative symptoms, such as upset and lacking of confidence, when the Internet is not available. The Internet search has made people dependent on search tools rather than their own memories.

Internet search trust refers to the people who trust the information obtained through search engines. Most people insist that they have benefited a lot from Internet search, as the Internet provides them with an effective way to find information and to gain skills that are important to their study, work as well as daily life, which in turn may facilitate the development of Internet search dependence.

External storage under Internet search describes the phenomenon that people are prone to remember where to access information instead of processing and storing the information by themselves, the so-called “Google effect” (7). The Internet, in general, has become an external storage for people to store information.

Given the ubiquitous presence of Internet, nowadays people can easily reach a huge amount of information with relatively little effort in a short period of time. With the frequent use of the Internet and the rapid advancement of technology, individuals are less willing to take notes or think independently and deeply, which in turn may result in a blind trust and firm reliance or dependence on Internet search. It has been evident that the over use of search engines leads to structural and functional brain changes (2, 8, 29). Although many researchers have noted that Internet search modifies psychological characteristics and facilitates some specific types of behaviors (e.g., seeking information), further study are required to address on those changes in more details.

In all, this study designed a questionnaire for measuring Internet search dependence and tested its reliability and validity through various analyses. The results of all analyses suggest that the QISD is an effective tool with good reliability and validity, which can serve as a practical tool for future studies to investigate the impacts of Internet search on people’s psychological characteristics. However, it should be aware that there are two limitations of this study: first, the current study provided a preliminary tool to measure the level of Internet search dependence, without providing norms or cutting lines. Thus, it cannot be used as a diagnostic tool. Second, the participants recruited for this research were all college students, and the majority was females, who may not be the representative of the general population. Future studies are needed to further test the QISD among various samples, such as adolescents.

## Ethics Statement

This research was approved by the Human Investigations Committee of Zhejiang Normal University. All participants in this research signed a written informed consent. No vulnerable populations were involved.

## Author Contributions

YW analyzed the data and wrote the first draft of the manuscript. HZ and YW performed the experiments and collected the data. GD conceived and designed the experiments. GD, LW, and JX revised and improved the manuscript.

## Conflict of Interest Statement

The authors declare that the research was conducted in the absence of any commercial or financial relationships that could be construed as a potential conflict of interest.

## Appendix

### The Questionnaire on Internet Search Dependence

Hello, we are researchers in the Department of Psychology of Zhejiang Normal University. We carry this investigation to explore how you search the Internet. This result will not be open to the public; please answer the questions honestly and seriously.

Please read the following information first:

1. Internet search means individuals using Internet search engines (for example, Google and Baidu) to find information on the Internet.
2. The apparatus where people use Internet search engines include computers, IPADs, smart phones, and other terminal sets.

Basic information:

1. Gender: Male/Female:
2. Age:
3. Grade:
4. Major:
5. Years since your first use Internet:
6. Years since your first Internet search:
7. The time you spend on Internet search every day: minutes:
8. Please estimate the times you search the Internet everyday:
9. The percentage of the time spent on using search engine takes____% of the total time online.

Please provide your answers according to the following criteria:

0. Never 1. Seldom 2. Sometimes 3. Usually 4. Always.

1. When facing debatable questions, I prefer to search Google/Baidu first rather than thinking by myself.
2. I won’t take any notes if I know I can search for the information on the Internet.
3. When I am asked a complex question, I usually try to abstract the key words from it.
4. When someone disagrees with my points, I usually search the Internet for the answer.
5. I think it is not necessary to remember a thing if we can find it from Internet search.
6. When somebody asks me a question, I will search the Internet for answers if I cannot figure it out immediately.
7. I can quickly abstract keywords from a sentence for a potential Internet search.
8. I will be upset if I cannot find an answer to a complex question through Internet search.
9. I think Internet search can satisfy daily needs, including learning and living.
10. I usually start to search online unconsciously when I am idle.
11. I am not confident about the answers in my memory if I cannot double-check them through Internet search.
12. I think we can find reliable information through Internet search.

Thanks for your sincere participation!

### The Dimensions of the Questionnaire on Internet Search Dependence

Habitual use of Internet search:

1. When facing debatable questions, I prefer to search Google/Baidu first rather than thinking by myself.
3. When I am asked a complex question, I usually try to abstract the key words from it.
4. When someone disagrees with my points, I usually search the Internet for the answer.
6. When somebody asks me a question, I will search the Internet for answers if I cannot figure it out immediately.
7. I can quickly abstract keywords from a sentence for a potential Internet search.

Withdrawal reaction:

8. I will be upset if I cannot find an answer to a complex question through Internet search.
11. I am not confident about the answers in my memory if I cannot double-check them through Internet search.

Internet search trust:

9. I think Internet search can satisfy daily needs, including learning and living.
10. I usually start to search online unconsciously when I am idle.
12. I think we can find reliable information through Internet search.

External storage under Internet search:

2. I won’t take any notes if I know I can search for the information on the Internet.
5. I think it is not necessary to remember a thing if we can find it from Internet search.
