# Supplementary material for: Development and Validation of a Self-reported Questionnaire for Measuring Internet Search Dependence
Source: Front Public Health. 2016 Dec 20;4:274. doi: 10.3389/fpubh.2016.00274 (PMC5167696; doi:10.3389/fpubh.2016.00274)
Supplement: Supplementary file 1 [file Presentation_1.PDF]

The questions that were asked during the interviews with 50 students

1. Do you frequently use the Internet search?

2. Do you use the Internet search when you have a problem?

3. Have you ever formed any habits during the process of Internet search?

4. What kind of habits do you develop?

5. When facing a debate question, do you search the web or book for the answer?

6. Is it been so long since you’ve looked for an answer from books last time?

7. Do you trust the information that on the Internet?

8. When you met a complex question, would you abstract keywords from the sentences?

9. Would you unconsciously open the search engines when you were bored?

10. Would you feel unsure of yourself when you could not check the information though Internet?

11. Do you think the Internet search can solve all problems about study and daily life?

12. Would you make a note of the answers that were found from the Internet?

13. Would you feel upset when you could not use Internet search?

14. Your attitude about Internet search? Positive or negative?

15. Advantages or disadvantages of Internet search?

The questions that were asked during the interviews with 100 students

The use of Internet search

(1) Years since your first use Internet:
(2) Years since your first Internet search:
(3) The time you spend on Internet search every day: minutes
(4) Please estimate the times you search the Internet everyday:
(5) The percentage of the time spent on using search engine takes____% of the total time online.

The questionnaire of Internet Search Dependence
Please provide your answers according to the following criteria:

0. Never 1. Seldom 2. Sometimes 3. Usually 4. Always

1. When facing debatable questions, I prefer to search Google/Baidu first.
2. I won’t take any notes if I know I can search for the information on the Internet.
3. When I am asked a complex question, I usually try to abstract the key words from it.
4. When someone disagrees with my points, I usually search the Internet for the answer.
5. I think it is not necessary to remember a thing if we can find it from Internet search.
6. When somebody asks me a question, I will search the Internet for answers if I cannot figure it out immediately.
7. I can quickly abstract keywords from a sentence for a potential Internet search.
8. I will be upset if I cannot find a complex question through Internet search.
9. I think Internet search can satisfy daily needs, including learning and living.
10. I usually start to search online unconsciously when I am idle.
11. I am not confident about the answers in my memory if I cannot double-check them through Internet search.
12. I think we can find reliable information through Internet search.

The four items that were removed before the analysis

1. It has been long time since I last looked for an answer from books.
2. I trust the information on the Internet more than that in my memory.
3. Once I have a problem, I will search it online for help.
4. I feel anxious in a context without the use of Internet Search.
